# Systemic Mastocytosis Treatment with Midostaurin: [^18^F]FDG PET/CT as a Potential Monitoring Tool for Therapy Outcome

**DOI:** 10.3390/diagnostics12030680

**Published:** 2022-03-10

**Authors:** Caroline Burgard, Florian Rosar, Fadi Khreish, Samer Ezziddin

**Affiliations:** Department of Nuclear Medicine, Saarland University, 66421 Homburg, Germany; caroline.burgard@uks.eu (C.B.); florian.rosar@uks.eu (F.R.); fadi.khreish@uks.eu (F.K.)

**Keywords:** systemic mastocytosis, FDG PET/CT, midostaurin, tyrosine kinase inhibitor, therapy outcome

## Abstract

We report the case of a 68-year-old patient with diagnosed systemic mastocytosis and histopathologically confirmed manifestations in the stomach and intestinal tract who underwent ^18^F-Fluorodeoxyglucose ([^18^F]FDG) positron-emission tomography/computed tomography (PET/CT) pre- and post-6-month therapy with midostaurin, an established tyrosine kinase inhibitor. Post-therapeutic [^18^F]FDG PET/CT showed decreased multifocal tracer uptake in the known lesions in the gastrointestinal tract, which was consistent with relief of the patient’s symptoms and decrease in serum tryptase level. [^18^F]FDG PET/CT may thus be considered a potential method for monitoring the outcome of midostaurin therapy in systemic mastocytosis.

The term mastocytosis describes a heterogeneous group of diseases, characterized by accumulation of clonal (neoplastic) mast cells in various organs [1]. The WHO classifies the disease into cutaneous mastocytosis, systemic mastocytosis (SM), and localized mast cell tumors on the basis of histomorphologic criteria, clinical parameters, and organ involvement [2]. It is a rare disease with an incidence of 4–5/1,000,000 per year [3]. Approximately 90% of patients are found to have a mutation in the KIT-D816V gene, which encodes a constitutively activated receptor tyrosine kinase that drives disease pathogenesis [4,5]. Several therapeutic approaches exist for the treatment of SM, including the purine analogue cladribine, interferon alpha, and the tyrosine kinase inhibitor imatinib [2], which show only a modest response to therapy [6,7]. In recent years, several studies have demonstrated the high efficacy and durable activity of therapy with midostaurin, a tyrosine kinase inhibitor, including KIT-D816V, in SM [8,9,10]. Serum levels of tryptase and mast cell involvement in the bone marrow are used as a standard to monitor the effect of therapy and correlate with the disease burden [1]. Except for determination of splenic volume to assess splenomegaly, there has been no suitable imaging to monitor the therapeutic effect of midostaurin [8]. The value of ^18^F-Fluorodeoxyglucose ([^18^F]FDG) whole body positron-emission tomography/computed tomography (PET/CT), which is an established method in staging and restaging of several malignant diseases, has not been investigated yet as a monitoring tool for mastocytosis. Only a few studies with a limited number of patients and case reports could demonstrate increased [^18^F]FDG uptake in mastocytosis manifestations, e.g., in cortical bone and bone marrow [11,12,13,14], which is a prerequisite for potential [^18^F]FDG PET/CT-based monitoring. 

In this case, a 68-year-old man with formerly diagnosed malignant melanoma was admitted to our hospital for progression control of known SM (subtype: SM with associated hematologic neoplasm), including gastrointestinal manifestations. Manifestations of mastocytosis in the mucosa of the stomach, duodenum, and colon were confirmed histologically. In the course of the disease, the patient was treated with cetirizine, fexofenadine, and clemastine, as well as budesonide and prednisolone. Nevertheless, he developed a malabsorption syndrome with persistent diarrhea and a concomitant body weight loss of 25 kg. After failure of these treatments, therapy with midostaurin (200 mg per day) was introduced. No relevant side effects such as nausea, vomiting, neutropenia, anemia, or thrombocytopenia occurred during the therapy. Within the follow-up care for malignant melanoma, the patient received a [^18^F]FDG PET/CT pre- and post-initiation of midostaurin therapy (Figure 1). After 6 months’ therapy (6 therapy cycles, respectively) with midostaurin, [^18^F]FDG PET/CT revealed a substantially decreased multifocal tracer uptake in the known lesions in the gastrointestinal tract. Diffuse moderate uptake in bone marrow was also slightly regressive, while splenomegaly remained similar. According to imaging, the patient’s clinical condition improved significantly, with diarrhea markedly regressing and body weight increasing by approximately 15 kg. Consistently, a decrease in serum tryptase level (615.3 μg/L pre- vs. 21.9 μg/L post-midostaurin treatment) was noted. The declining [^18^F]FDG uptake of histologically confirmed manifestations of SM in the gastrointestinal tract during the course thus most likely represents a response to therapy with midostaurin. 

To the best of our knowledge, the reported case represents the first evidence that [^18^F]FDG PET/CT may be a potential imaging modality to monitor the therapeutic effect of midostaurin in SM. A key advantage of [^18^F]FDG PET/CT imaging over other imaging modalities such as CT, MRI, or ultrasound in therapy monitoring of midostaurin in SM may be the exclusive ability to estimate disease activity based on glucose metabolism and, thus, therapy response. This case report may provide a rational starting point for further systematic studies investigating the value of [^18^F]FDG PET/CT in clinical management and monitoring of SM.

## Figures and Tables

**Figure 1:**
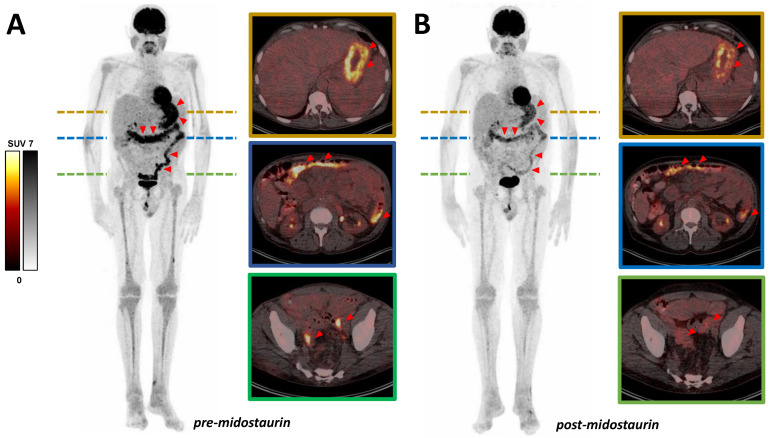
Declining uptake can be seen in presented MIP (maximum intensity projection) and exemplary transversal slices of [^18^F]FDG PET/CT scan in gastrointestinal mastocytosis manifestations. (**A**) Baseline (blood glucose level: 87 mg/dL; hemoglobin: 11.2 g/dL) and (**B**) after 6 months’ therapy with midostaurin (blood glucose level: 103 mg/dL; hemoglobin: 11.5 g/dL). Exemplary SUV_peak_ values (pre- vs. post-midostaurin treatment): in stomach 7.2 vs. 5.8 and in colon 6.4 vs. 3.4. Diffuse uptake in bone marrow was also slightly regressive (exemplary in the left tibia SUV_peak_ 2.3 vs. 1.9), while splenomegaly remained similar. Red triangles exemplarily point to gastrointestinal mastocytosis manifestations.

## Data Availability

The datasets used and analyzed in this paper are available from the corresponding author on reasonable request.
